# Risk factors of postoperative complications and their effect on survival after laparoscopic gastrectomy for gastric cancer

**DOI:** 10.1002/ags3.12780

**Published:** 2024-02-24

**Authors:** Vo Duy Long, Dang Quang Thong, Tran Quang Dat, Doan Thuy Nguyen, Nguyen Viet Hai, Ho Le Minh Quoc, Nguyen Vu Tuan Anh, Nguyen Lam Vuong, Nguyen Hoang Bac

**Affiliations:** ^1^ Gastro‐intestinal Surgery Department, University Medical Center University of Medicine and Pharmacy at Ho Chi Minh City Ho Chi Minh City Vietnam; ^2^ Department of General Surgery, Faculty of Medicine University of Medicine and Pharmacy at Ho Chi Minh City Ho Chi Minh City Vietnam; ^3^ Department of Medical Statistics and Informatics, Faculty of Public Health University of Medicine and Pharmacy at Ho Chi Minh City Ho Chi Minh City Vietnam

**Keywords:** gastric cancer, laparoscopic distal gastrectomy, laparoscopic gastrectomy, laparoscopic total gastrectomy, postoperative complications

## Abstract

**Background:**

The association between postoperative complications and long‐term survival after laparoscopic gastrectomy (LG) for gastric cancer (GC) remains uncertain. This study aimed to determine the incidence and risk factors of postoperative complications and evaluate their impact on survival outcomes in patients undergoing LG.

**Methods:**

A retrospective study was conducted on 621 patients who underwent LG for gastric adenocarcinoma between March 2015 and December 2021. Postoperative complications were classified according to the Clavien–Dindo classification, with major complications defined as Grade III or higher. Logistic regression models with stepwise backward procedure were used to identify risk factors for complications. To assess the impact of postoperative complications on survival, uni‐ and multi‐variable Cox proportional hazard models were used for overall survival (OS) and disease‐free survival (DFS).

**Results:**

Overall rate of postoperative complications was 17.6% (109 patients); 33 patients (5.3%) had major complications. Independent risk factors for major complications were Charlson comorbidities index (OR [95% CI], 1.87 [1.09–3.12], *p*‐value = 0.018 for each one score increase), and type of anastomosis (OR [95% CI], 0.28 [0.09–0.91], *p*‐value = 0.029 when comparing Billroth II with Billroth I). Multivariable analysis identified major complications as an independent prognostic factor to reduce OS (HR [95% CI], 2.32 [1.02–5.30], *p*‐value = 0.045) and DFS (HR [95% CI], 2.63 [1.37–5.06], *p*‐value = 0.004). Other prognostic factors for decreased survival outcomes were tumor size, presence of invasive lymph nodes, and T4a stage.

**Conclusions:**

Major complications rate of LG for GC was approximately 5.3%. Charlson comorbidities index and type of anastomosis were identified as risk factors for major postoperative complications. Major complications were demonstrated to pose adverse impact on survival outcomes.

## Graphical abstract

Postoperative complications after laparoscopic gastrectomy are acceptable. Charlson comorbidities index was an independent risk factor for complications. Major postoperative complications adversely affect overall and disease‐free survival.

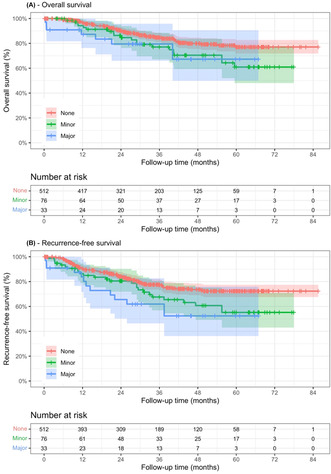

## 1 INTRODUCTION

Gastric cancer (GC) is a prevalent malignancy and a leading cause of cancer‐related mortality, representing a significant global health burden across the world.^1^ Despite notable advancements in multimodality treatment and targeted therapy, gastrectomy remains the primary treatment option for this disease. Laparoscopic radical gastrectomy is commonly employed for early GC and is increasingly being utilized for advanced GC. This procedure has become the standard approach for proficient surgeons. However, surgeons trained in low‐volume centers or under less experienced laparoscopic gastrectomy (LG) instructors may encounter technical challenges.^2^, ^3^, ^4^, ^5^, ^6^

Radical gastrectomy plays a crucial role in improving survival outcomes of GC patients. However, this surgical technique is intricate and challenging, leading to a high incidence of postoperative complications. Achieving proper lymph node dissection and R0 resection are essential components of radical gastrectomy. Despite advancements in surgical techniques and perioperative care, the occurrence of postoperative morbidity and mortality remains relatively high, ranging from 10% to 25%.^2^, ^3^, ^4^, ^5^, ^7^, ^8^, ^9^ Major postoperative complications, such as abdominal abscess, bleeding, anastomotic leak, pneumonia, can contribute to increased treatment costs, prolonged hospitalization, and an elevated risk of recurrence and worse long‐term survival. Several studies have suggested the detrimental impact of postoperative complications on the oncological prognosis of GC patients, including decreased overall survival (OS) and disease‐free survival (DFS).^7^, ^10^, ^11^, ^12^, ^13^, ^14^, ^15^, ^16^

While surgical techniques and surgeon expertise have improved, managing postoperative complications remains a persistent challenge. The Clavien–Dindo classification system serves as a universally accepted standard for assessing postoperative complications.^2^, ^8^, ^9^, ^14^ The rate and severity of postoperative morbidity can be utilized as a metric to evaluate the quality of surgery. Identifying preoperative risk factors is crucial for mitigating postoperative complications.^7^, ^17^, ^18^ Various studies have investigated the risk factors associated with post‐LG complications; however, the findings have been heterogenous.^7^, ^10^, ^14^, ^15^, ^16^ Recent research has also suggested that the occurrence of postoperative complications, specifically anastomotic leak, negatively impacts long‐term survival outcomes.^19^, ^20^, ^21^, ^22^ Nonetheless, the association between postoperative complications and long‐term survival after LG for GC remains a subject of debate.

In this study, we aimed to examine the incidence of postoperative complications following LG and lymphadenectomy using the Clavien–Dindo classification system. Additionally, we sought to analyze the risk factors associated with these complications and the impact of complications on survival outcomes in patients with GC within a single institution.

## 2 PATIENTS AND METHODS

### 2.1 Study design and population

This retrospective study was conducted at the Gastro‐Intestinal Surgery Department of the University Medical Center Ho Chi Minh City, a referral hospital in Southern Vietnam. We included all patients who underwent LG for GC between March 2015 and December 2021.

The inclusion criteria for the study were as follows: (i) histologically confirmed adenocarcinoma of the stomach; (ii) surgical staging of sT1–T4a, N0–3, M0 based on the 7th edition of the American Joint Committee on Cancer/Union Internationale Contre le Cancer (AJCC/UICC) staging system. Exclusion criteria encompassed the following: (i) proximal gastrectomy; (ii) intraoperatively detected bulky lymph nodes; (iii) inadequate lymphadenectomy (D0, D1); (iv) presence of macroscopic residual tumor (R2); (v) concurrent cancer or a history of previous other cancers; (vi) previous gastrectomy, (vii) palliative gastrectomy; (viii) complications such as bleeding or perforation requiring emergency gastrectomy, (ix) lost to follow‐up within 30 days after surgery.

We started performing laparoscopic proximal gastrectomy with double flap reconstruction in 2018 and did only four cases for early GC. Therefore, we excluded this procedure in this study to avoid bias.

All patients included in the study were confirmed to have GC through preoperative endoscopic biopsy and histological analysis. Additionally, abdominal computed tomography (CT) scan was performed to assess the stage and location of the tumor. The indication for LG was cT1b–T4a, N0–3, M0, and cT1aN0M0 with a failed endoscopic submucosal resection.

### 2.2 Surgical techniques

Surgical techniques applied in this study followed the principles of laparoscopic distal gastrectomy (LDG) and laparoscopic total gastrectomy (LTG), in accordance with the Japanese guidelines for the extent of gastrectomy and D2 lymphadenectomy.^23^ During the procedures, total omentectomy was performed in all cases. The anastomosis was performed using intracorporeal techniques with a linear stapler. In LDG, the anastomosis methods included Billroth‐I, Billroth‐II, and Roux‐en‐Y, while in LTG, Roux‐en‐Y was utilized for all cases. Mobilization and dissection were conducted using an ultrasonic scalpel. Routine peritoneal lavage cytology was not performed. All operations were performed by three experienced surgeons, each with a track record of over 200 standard open and laparoscopic gastrectomies for GC. We have performed laparoscopic gastrectomy for early GC since 2008 and for locally advanced GC since 2012. The technique of LG and lymph node dissection had been standardized for advanced GC since 2013. All surgeons in this study achieved an adequate learning curve since 2013.

Prophylactic antibiotics were administered as a routine practice. Abdominal drains were routinely inserted and removed within 2–3 days after surgery. The use of nasogastric tube was not standard practice. Since 2017, the enhanced recovery after surgery (ERAS) program has been implemented for all LDG cases.

### 2.3 Patient evaluation and follow‐up

Patient characteristics including gender, age, body mass index (BMI), previous abdominal surgery, American Society of Anesthesiologists physical status (ASA‐PS) classification, presence of preoperative anemia, tumor location, tumor size, receipt of preoperative chemotherapy, operating time, blood loss, type of surgery, type of anastomosis, level of lymphadenectomy, differentiation classification, presence of invasive lymph nodes, tumor stage, and Charlson comorbidities index^24^ were collected to assess the risk factors for postoperative complications.

The follow‐up schedule adhered to the Japanese guidelines for GC management. Patients with pathological stage II or higher received adjuvant chemotherapy with either capecitabine and oxaliplatin (XELOX) or fluorouracil, leucovorin, and oxaliplatin (FOLFOX) or TS‐ONE®. During the first 2 years after surgery, patients were followed up every 3 months, followed by biannual visits for the subsequent 3 years, and then annual visits. During each follow‐up visit, patients underwent physical examinations, laboratory blood tests, and abdominal ultrasonography. CT scans were conducted every 6 months during the first 3 years and annually thereafter. Endoscopy was performed annually. In cases where patients exhibited suspected symptoms or signs of recurrence or metastasis, CT scans and/or endoscopy were performed to evaluate and confirm the presence of such conditions, regardless of the scheduled follow‐up appointments. The length of follow‐up was defined as the duration from the date of surgery to the final follow‐up date in December 2022 or the date of death. Cancer recurrence was diagnosed based on radiologic or histological evidence of disease.

### 2.4 Outcomes

Postoperative complications were evaluated within 30 days after LG using the Clavien–Dindo classification system, which consists of five main grades.^25^ Grade I complications do not require any pharmacological treatment or intervention. Grade II complications necessitate pharmacological therapy. Grade III complications require surgical, endoscopic, or radiological intervention under regional/local anesthesia (III‐a) or general anesthesia (III‐b). Grade IV complications indicate life‐threatening conditions that require immediate care/intensive care unit management with either single organ dysfunction (IV‐a) or multiple organ dysfunctions (IV‐b). Grade V is assigned in the event of death. Major complications were defined as Grade III or higher.

Diagnosis of anastomotic leak or duodenal stump leak was based on clinical symptoms and signs of peritonitis, along with radiologic examinations demonstrating contrast leakage into abdominal cavity or confirmed during reoperation. Abdominal abscess was confirmed by the presence of a collection of pus, either through percutaneous drainage or during reoperation. Bleeding complications were defined by the requirement of a blood transfusion (two units or more) and clinical symptoms indicating intra‐abdominal or gastrointestinal hemorrhage. Pancreatic fistula was diagnosed when any measurable volume of fluid was observed in the drain after the third postoperative day with an amylase content exceeding three times the serum level. Wound infection was diagnosed when purulent exudate was found in the wound, accompanied by positive bacterial culture. Postoperative mortality was defined as any death occurring during the hospital stay following surgery. Pneumonia was confirmed by clinical pyrexia and infiltration on the chest X‐ray.

Long‐term oncological outcomes were assessed in terms of OS and DFS during the follow‐up period. OS represents the duration from surgery to death from all causes, while DFS measures the length of time from surgery to the detection of recurrence or metastasis, or death from any cause. If no events (recurrence, metastasis, or death) occurred, OS and DFS were censored at the last follow‐up time.

### 2.5 Statistical analysis

To investigate risk factors of postoperative complication, we conducted separate analyses for overall complication and major complication, both of which were binary variables (Yes/No). Minor complications were included in the “Yes” group for the overall complications but were included in the “No” group for the major complication analysis. Univariable analysis was performed using two‐sample t‐test for normally distributed numeric variables, the Wilcoxon rank‐sum test for non‐normally distributed numeric variables, and Fisher's exact test for categorical variables. Multivariable analysis was conducted using logistic regression models with a stepwise backward procedure to identify independent risk factors of postoperative complications.

Survival outcomes were presented using Kaplan–Meier plots stratified by the level of complications (no complications, minor complications, and major complications). To assess the impact of postoperative complications on survival, uni‐ and multivariable Cox proportional hazard models were used for OS and DFS analyses, including complication with three levels and other potential confounding factors.

All analyses were performed using R statistical software version 4.1.0. Two‐sided tests were used for all analyses, and a *p*‐value less than 0.05 was considered statistically significant. The results from the models were reported as odds ratios (ORs) for logistic regression models and hazard ratios (HRs) for Cox models, along with their corresponding 95% confidence intervals (CIs).

## 3 RESULTS

**FIGURE 1:**
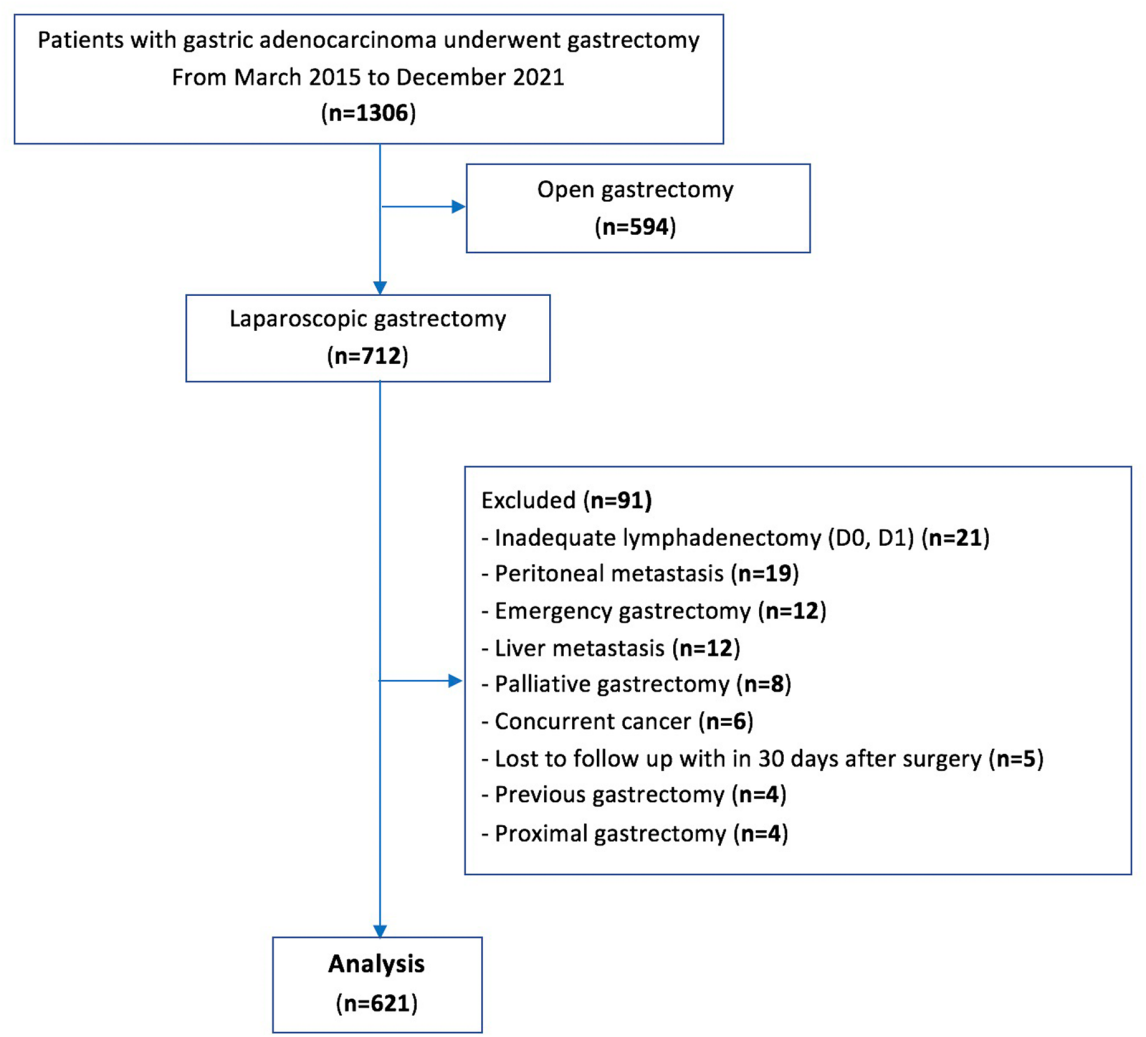
Flowchart of patient selection.

Between March 2015 and December 2021, a total of 712 patients with gastric adenocarcinoma underwent LG in our center. After excluding 91 patients, 621 patients were included in this study (Figure 1). The distribution of patients by period was as follows: 2015–2017—177 patients; 2018–2019—226 patients; and 2020–2021—218 patients.

### 3.1 Clinical characteristics

Table 1 presented the patients' clinical characteristics. The mean age was 59.5 ± 11.9 years, and 72.2% were of normal weight. The most common comorbidity was hypertension (24%), followed by diabetes (10.6%) and ischemic heart disease (5.8%). Forty‐four patients (7.1%) had a history of prior abdominal surgery. Most patients (87.6%) were classified as ASA‐PS classification I or II.

**TABLE 1:** Clinical characteristics.

	All patients (*N* = 621)	No complication (*N* = 512)	Minor complication (*N* = 76)	Major complication (*N* = 33)
Age (years)	59.5 ± 11.9	59.2 ± 12.0	62.0 ± 11.8	59.9 ± 11.3
Sex male	381 (61.4)	313 (61.1)	49 (64.5)	19 (57.6)
BMI (kg/m^2^)	21.3 ± 2.9	21.2 ± 2.9	21.7 ± 3.0	22.1 ± 3.6
Nutritional status				
Underweight (BMI <18.5)	111 (18.0)	93 (18.2)	12 (16.2)	6 (18.8)
Normal weight (BMI: 18.5–24.9)	446 (72.2)	377 (73.6)	50 (67.6)	19 (59.4)
Overweight (BMI: 25–30)	57 (9.2)	40 (7.8)	11 (14.9)	6 (18.8)
Obese (BMI >30)	4 (0.6)	2 (0.4)	1 (1.4)	1 (3.1)
Hypertension	149 (24.0)	115 (22.5)	21 (27.6)	13 (39.4)
Type‐2 diabetes	66 (10.6)	52 (10.2)	5 (6.6)	9 (27.3)
Prior abdominal surgery	44 (7.1)	35 (6.8)	6 (7.9)	3 (9.1)
Ischemic heart disease	36 (5.8)	28 (5.5)	6 (7.9)	2 (6.1)
COPD	14 (2.3)	12 (2.3)	2 (2.6)	0 (0.0)
Prior stroke	7 (1.1)	4 (0.8)	1 (1.3)	2 (6.1)
Chronic kidney disease	6 (1.0)	5 (1.0)	0 (0.0)	1 (3.0)
Cirrhosis	4 (0.6)	3 (0.6)	0 (0.0)	1 (3.0)
Charlson comorbidities index	3.8 ± 1.3	3.7 ± 1.3	3.9 ± 1.4	4.1 ± 1.1
ASA‐PS classification				
I	359 (57.8)	310 (60.5)	35 (46.1)	14 (42.4)
II	185 (29.8)	141 (27.5)	31 (40.8)	13 (39.4)
III	77 (12.4)	61 (11.9)	10 (13.2)	6 (18.2)
Preoperative anemia	107 (17.2)	79 (15.4)	22 (28.9)	6 (18.2)
Tumor location				
Upper third	25 (4.0)	15 (2.9)	7 (9.2)	3 (9.1)
Middle third	119 (19.2)	105 (20.5)	8 (10.5)	6 (18.2)
Lower third	422 (68.0)	343 (67.0)	59 (77.6)	20 (60.6)
Upper and middle third	14 (2.3)	11 (2.1)	1 (1.3)	2 (6.1)
Middle and lower third	35 (5.6)	33 (6.4)	1 (1.3)	1 (3.0)
Whole stomach	6 (1.0)	5 (1.0)	0 (0.0)	1 (3.0)
Tumor size (cm)	3.6 ± 1.8	3.6 ± 1.8	3.4 ± 1.6	4.1 ± 1.9
Preoperative chemotherapy	18 (2.9)	16 (3.1)	1 (1.3)	1 (3.0)
Operating time (min)	214 ± 56	211 ± 54	220 ± 58	241 ± 72
Blood loss (mL)				
<100	372 (59.9)	316 (61.7)	39 (51.3)	17 (51.5)
≥100	249 (40.1)	196 (38.3)	37 (48.7)	16 (48.5)
Type of surgery				
Distal gastrectomy	530 (85.3)	443 (86.5)	65 (85.5)	22 (66.7)
Total gastrectomy	91 (14.7)	69 (13.5)	11 (14.5)	11 (33.3)
Level of lymphadenectomy				
D1+	30 (4.8)	23 (4.5)	5 (6.6)	2 (6.1)
D2	564 (90.8)	470 (91.8)	66 (86.8)	28 (84.8)
D2+	27 (4.3)	19 (3.7)	5 (6.6)	3 (9.1)
Type of anastomosis				
Billroth II	304 (49.0)	264 (51.6)	33 (43.4)	7 (21.2)
Roux‐en‐Y	230 (37.0)	178 (34.8)	32 (42.1)	20 (60.6)
Billroth I	87 (14.0)	70 (13.7)	11 (14.5)	6 (18.2)
Postop hospital length of stay (days)	8 (7; 9)	7 (7; 8)	10 (8; 15)	12 (10; 19)
Differentiation classification				
Well differentiated	48 (7.7)	41 (8.0)	5 (6.6)	2 (6.1)
Moderately differentiated	222 (35.7)	179 (35.0)	32 (42.1)	11 (33.3)
Poorly differentiated	264 (42.5)	218 (42.6)	32 (42.1)	14 (42.4)
Signet ring cell	87 (14.0)	74 (14.5)	7 (9.2)	6 (18.2)
Invasive lymph nodes	344 (55.4)	279 (54.5)	49 (64.5)	16 (48.5)
R0 surgery	610 (98.2)	501 (97.9)	76 (100.0)	33 (100.0)
T stage				
T1	118 (19.0)	104 (20.3)	8 (10.5)	6 (18.2)
T2	96 (15.5)	85 (16.6)	5 (6.6)	6 (18.2)
T3	23 (3.7)	21 (4.1)	2 (2.6)	0 (0.0)
T4a	384 (61.8)	302 (59.0)	61 (80.3)	21 (63.6)
Required adjuvant chemotherapy	512 (82.4)	414 (80.9)	71 (93.4)	27 (81.8)
Received adjuvant chemotherapy				
None	111 (21.7)	85 (20.5)	16 (22.5)	10 (37.0)
Incomplete	56 (10.9)	46 (11.1)	8 (11.3)	2 (7.4)
Complete	345 (67.4)	283 (68.4)	47 (66.2)	15 (55.6)
Length of follow‐up (months)	30 (19; 49)	30 (19; 47)	34 (20; 59)	29 (12; 46)

*Note*: Statistical summary is mean ± standard deviation, median (25th; 75th percentiles), or *n* (%).

Abbreviations: ASA‐PS, The American Society of Anesthesiologists physical status; BMI, body mass index; COPD, chronic obstructive pulmonary disease.

Regarding tumor characteristics, the most common location of the tumors was the lower third of the stomach (68%), followed by the middle third. The mean tumor size was 3.6 cm. Most patients (61.8%) were diagnosed at the locally advanced stage with serosa invasion (T4a). Preoperative chemotherapy was administered to 2.9% of the patients. LDG was performed in 530 patients (85.3%), while LTG was performed in 91 patients (14.7%). The Roux‐en‐Y reconstruction method was utilized in all 91 LTG cases and in 139 LDG cases. The most used reconstruction method for LDG was the Billroth‐II. D2 lymphadenectomy was performed in 564 cases (90.8%), and R0 resection was achieved in 610 patients (98.2%). The mean operating time was 214 min, and 249 patients (40.1%) experienced blood loss of 100 mL or more.

Among the patients, the length of postoperative stay was relatively longer in the major group (average: 12 [10;19] days) compared to the minor complication group (average: 10 [8;15] days) and no complication group (average: 7 [7;8] days). Additionally, 512 patients (82.4%) were indicated for adjuvant chemotherapy. However, only 345 patients (67.4%) received a complete schedule of adjuvant chemotherapy. The proportion of patients who received complete adjuvant chemotherapy was lower in the major complication group (55.6%) compared to the no complication group (68.4%) and minor complication group (66.2%).

### 3.2 Postoperative complications

Postoperative complications were observed in 109 patients (17.6%). Among those, 76 patients (12.2%) had minor complications, while 33 patients (5.3%) experienced major complications. The most common complications were pneumonia (4.5%), paralytic ileus (3.4%), abdominal abscess (2.7%), intra‐abdominal bleeding (2.3%), and pancreatic leak (2.3%). In the major complication group, the most frequent complications were pneumonia (36.4%), intra‐abdominal bleeding (33.3%), and abdominal abscess (27.3%) (Table S1). Three patients (0.5%) died during their hospital stay, with two cases attributed to severe septic shock caused by anastomotic leak and one case due to severe bleeding.

For anastomosis‐related complications after laparoscopic distal gastrectomy, six patients experienced major complications related to Billroth I anastomosis, including intra‐abdominal abscess (three patients), anastomotic stenosis (two patients), and major anastomotic leak (one patient). However, no anastomosis related complications were observed in the seven patients who experienced major postoperative complications in the Billroth II anastomosis group.

### 3.3 Risk factors for postoperative complications

In the univariable analyses, factors including preoperative anemia, tumor location in the upper‐third of the stomach, longer operating time, type of anastomosis, and more advanced T stage were found to significantly increase the risk of overall complications. The results from the multivariable analyses showed that BMI (OR [95% CI], 1.10 [1.03–1.19], *p*‐value = 0.009 for each one kg/m^2^ increase), Charlson comorbidities index (OR [95% CI], 1.19 [1.00–1.40], *p*‐value = 0.047 for each one score increase), tumor location (OR [95% CI], 0.40 [0.17–0.82], *p*‐value = 0.02 when comparing middle third/middle and lower third with lower third only), tumor size (OR [95% CI], 0.83 [0.71–0.97], *p*‐value = 0.024 for each 1 cm increase), and advanced T stage (OR [95% CI], 2.58 [1.52–4.51], *p*‐value =<0.001 when comparing T4a with T1–T3) were identified as independent risk factors for overall postoperative complications (Table 2).

**TABLE 2:** Uni‐ and multivariable analysis of risk factors for overall complications.

	Univariable analysis			Multivariable analysis		
	No (*N* = 512)	Yes (*N* = 109)	*p*‐value	OR	95% CI	*p*‐value
Age (years)	59.2 ± 12.0	61.3 ± 11.6	0.091			
Sex			0.829			
Male	313 (82.2)	68 (17.8)				
Female	199 (82.9)	41 (17.1)				
BMI (kg/m^2^)	21.2 ± 2.9	21.8 ± 3.2	0.141	1.10	1.03, 1.19	0.009
Charlson comorbidities index	3.7 ± 1.3	4.0 ± 1.3	0.090	1.19	1.00, 1.40	0.047
ASA‐PS classification			0.425			
I‐II	451 (82.9)	93 (17.1)				
III	61 (79.2)	16 (20.8)				
Preoperative anemia			0.017			
No	433 (84.2)	81 (15.8)		1	Ref	
Yes	79 (73.8)	28 (26.2)		1.72	0.98, 2.96	0.054
Tumor location			0.003			
Upper third/upper and middle third/whole stomach	31 (68.9)	14 (31.1)		1.02	0.28, 3.72	0.977
Middle third/middle and lower third	138 (89.6)	16 (10.4)		0.40	0.17, 0.82	0.020
Lower third only	343 (81.3)	79 (18.7)		1	Ref	
Tumor size (cm)	3.6 ± 1.8	3.6 ± 1.7	0.941	0.83	0.71, 0.97	0.024
Preop chemotherapy			0.753			
No	496 (82.3)	107 (17.7)				
Yes	16 (88.9)	2 (11.1)				
Operating time (min)	211 ± 54	226 ± 63	0.020			
Blood loss (mL)			0.053			
<100	316 (84.9)	56 (15.1)				
≥100	196 (78.7)	53 (21.3)				
Type of surgery			0.075			
Distal gastrectomy	443 (83.6)	87 (16.4)		1	Ref	
Total gastrectomy	69 (75.8)	22 (24.2)		2.75	0.91, 8.35	0.069
Type of anastomosis			0.014			
Billroth I	70 (80.5)	17 (19.5)				
Billroth II	264 (86.8)	40 (13.2)				
Roux en y	178 (77.4)	52 (22.6)				
Level of lymphadenectomy			0.139			
D1+	23 (76.7)	7 (23.3)				
D2	470 (83.3)	94 (16.7)				
D2+	19 (70.4)	8 (29.6)				
Differentiation classification			0.785			
Well differentiated	41 (85.4)	7 (14.6)				
Moderately differentiated	179 (80.6)	43 (19.4)				
Poorly differentiated	218 (82.6)	46 (17.4)				
Signet ring cell	74 (85.1)	13 (14.9)				
Invasive lymph nodes			0.341			
No	233 (84.1)	44 (15.9)				
Yes	279 (81.1)	65 (18.9)				
R0 surgery			0.227			
No	11 (100.0)	0 (0.0)				
Yes	501 (82.1)	109 (17.9)				
T stage			0.002			
T1–T3	210 (88.6)	27 (11.4)		1	Ref	
T4a	302 (78.6)	82 (21.4)		2.58	1.52, 4.51	<0.001

*Note*: Statistical summary is mean ± standard deviation, median (25th; 75th percentiles), or *n* (%).

Abbreviations: ASA‐PS, The American Society of Anesthesiologists physical status; BMI, body mass index; CI, confidence interval; HR, hazard ratio.

Regarding the major postoperative complications, the results of the univariate analysis revealed that operating time, type of surgery, and type of anastomosis were associated with a higher rate of major complications. Furthermore, the multivariable analysis identified Charlson comorbidities index (OR [95% CI], 1.87 [1.09–3.12], *p*‐value = 0.018 for each one score increase), and type of anastomosis (OR [95% CI], 0.28 [0.09–0.91], *p*‐value = 0.029 when comparing Billroth II with Billroth I) as independent risk factors for the development of major complications (Table 3).

**TABLE 3:** Uni‐ and multivariable analysis of risk factors for major complications.

	Univariable analysis			Multivariable analysis		
	No (*N* = 588)	Yes (*N* = 33)	*p*‐value	OR	95% CI	*p*‐value
Age (years)	59.5 ± 12.0	59.9 ± 11.3	0.559	0.95	0.89, 1.01	0.084
Sex			0.714			
Male	362 (95.0)	19 (5.0)				
Female	226 (94.2)	14 (5.8)				
BMI (kg/m^2^)	21.3 ± 2.9	22.1 ± 3.6	0.266			
Charlson comorbidities index	3.8 ± 1.3	4.1 ± 1.1	0.111	1.87	1.09, 3.12	0.018
ASA‐PS classification			0.281			
I‐II	517 (95.0)	27 (5.0)				
III	71 (92.2)	6 (7.8)				
Preoperative anemia			0.815			
No	487 (94.7)	27 (5.3)				
Yes	101 (94.4)	6 (5.6)				
Tumor location			0.069			
Upper third/upper and middle third/whole stomach	39 (86.7)	6 (13.3)				
Middle third/middle and lower third	147 (95.5)	7 (4.5)				
Lower third only	402 (95.3)	20 (4.7)				
Tumor size (cm)	3.6 ± 1.7	4.1 ± 1.9	0.118			
Preop chemotherapy			1			
No	571 (94.7)	32 (5.3)				
Yes	17 (94.4)	1 (5.6)				
Operating time (min)	212 ± 55	241 ± 72	0.022	1.17	0.98, 1.38	0.066
Blood loss (mL)			0.362			
<100	355 (95.4)	17 (4.6)				
≥100	233 (93.6)	16 (6.4)				
Type of surgery			0.004			
Distal gastrectomy	508 (95.8)	22 (4.2)				
Total gastrectomy	80 (87.9)	11 (12.1)				
Type of anastomosis			0.002			
Billroth I	81 (93.1)	6 (6.9)		1	Ref	
Billroth II	297 (97.7)	7 (2.3)		0.28	0.09, 0.91	0.029
Roux en y	210 (91.3)	20 (8.7)		0.84	0.32, 2.53	0.746
Level of lymphadenectomy			0.186			
D1+	28 (93.3)	2 (6.7)				
D2	536 (95.0)	28 (5.0)				
D2+	24 (88.9)	3 (11.1)				
Differentiation classification			0.910			
Well differentiated	46 (95.8)	2 (4.2)				
Moderately differentiated	211 (95.0)	11 (5.0)				
Poorly differentiated	250 (94.7)	14 (5.3)				
Signet ring cell	81 (93.1)	6 (6.9)				
Invasive lymph nodes			0.473			
No	260 (93.9)	17 (6.1)				
Yes	328 (95.3)	16 (4.7)				
R0 surgery			1			
No	11 (100.0)	0 (0.0)				
Yes	577 (94.6)	33 (5.4)				
T stage			1			
T1–T3	225 (94.9)	12 (5.1)				
T4a	363 (94.5)	21 (5.5)				

*Note*: Statistical summary is mean ± standard deviation, median (25th; 75th percentiles), or *n* (%).

Abbreviations: ASA‐PS, The American Society of Anesthesiologists physical status; BMI, body mass index; CI, confidence interval; HR, hazard ratio; Ref, reference.

### 3.4 Postoperative complications and survivals

**FIGURE 2:**
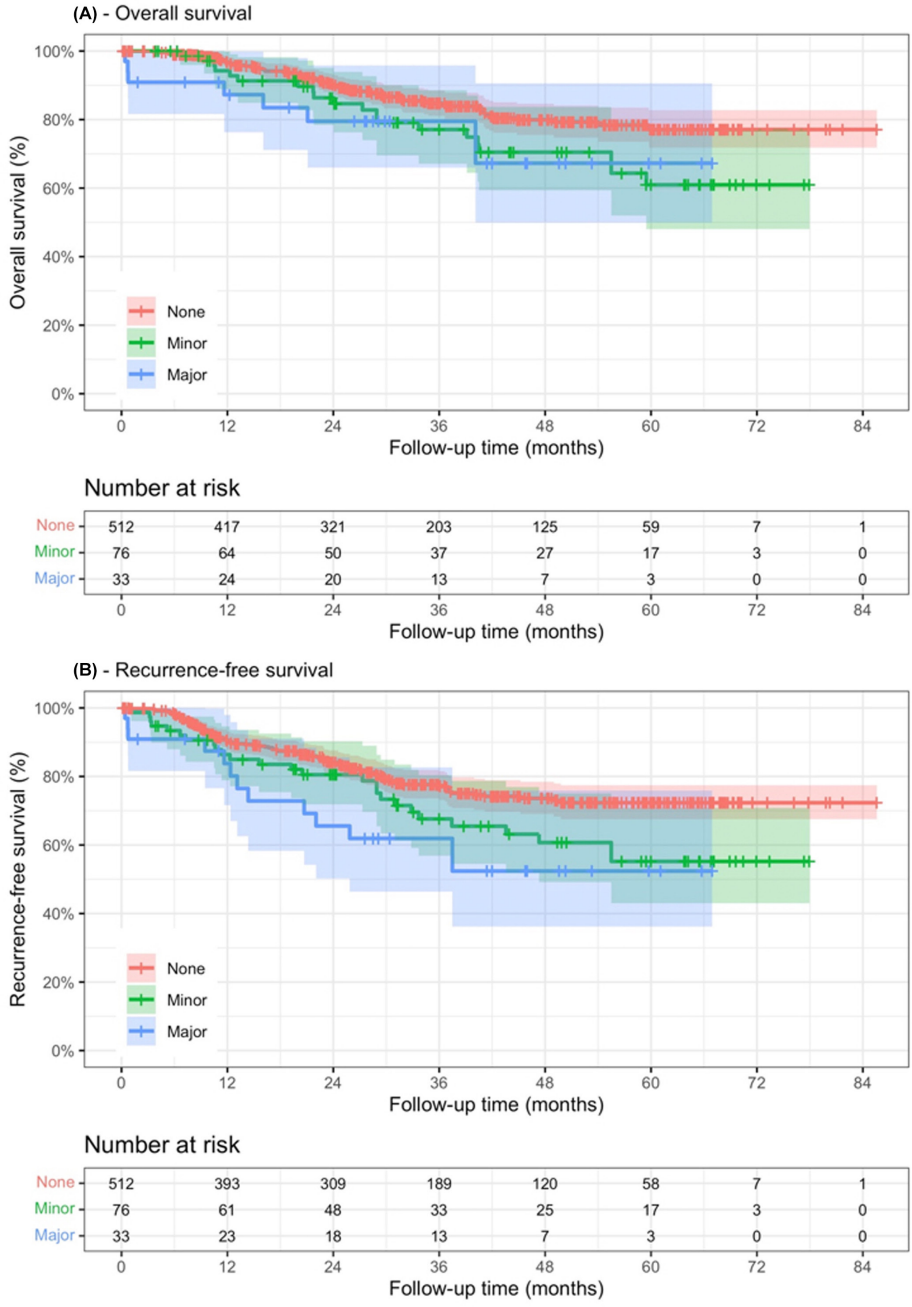
Kaplan–Meier survival curves for overall survival and disease‐free survival stratified by complication groups.

During a median follow‐up period of 30 months, disease progression occurred in 104 patients, and 99 patients died. The survival rate of both the minor and major complication groups appeared to be lower compared to the group with no complications (Figure 2). The 3‐year OS rates (excluded patients with preoperative chemotherapy) for the no complication, minor complication, and major complication groups were 86%, 77%, and 79%, respectively. The 3‐year DFS rates were 78%, 67%, and 61% for the respective groups (Table S2). The most common type of recurrence was peritoneal (62 cases, 59.6%), followed by hematogenous (15 cases, 14.4%) (Table S3).

Regarding risk factor for survival, the univariate analysis indicated that age, BMI, Charlson comorbidities index, preoperative anemia, tumor location, tumor size, type of surgery, level of lymphadenectomy, presence of invasive lymph nodes, T stage, and minor and major complications were all significantly associated with the OS rate. Additionally, with the univariable analysis, BMI, preoperative anemia, tumor location, tumor size, type of surgery, type of anastomosis, presence of invasive lymph nodes, R0 surgery, T stage, and minor and major complications were correlated with a decreased DFS rate.

The major complication was identified as an independent risk factor for decreasing both OS and DFS (HR [95% CI], 2.32 [1.02–5.30], *p*‐value = 0.045 for OS and 2.63 [1.37–5.06], *p*‐value = 0.004 for DFS) in the multivariable analysis.

Regarding the other prognostic factors for decreased survival outcomes, the results of the multivariable analysis demonstrated that tumor size, presence of invasive lymph nodes, and T4a stage were also independent risk factors of decreased OS and DFS (Table 4, Table 5).

**TABLE 4:** Uni‐ and multivariable analyses of risk factors of overall survival (excluded patients with preoperative chemotherapy).

	Univariable analysis			Multivariable analysis		
	HR	95% CI	*p*‐value	HR	95% CI	*p*‐value
Age (per 10 years increase)	1.30	1.09, 1.55	0.004	1.00	0.63, 1.60	0.987
Sex						
Male	—	—		—	—	
Female	1.37	0.91, 2.07	0.130	1.09	0.70, 1.69	0.702
BMI (kg/m^2^)	0.92	0.85, 0.99	0.031	0.95	0.88, 1.03	0.236
Charlson comorbidities Index	1.24	1.06, 1.44	0.006	1.35	0.84, 2.17	0.220
ASA‐PS classification						
I‐II	—	—		—	—	
III	1.23	0.67, 2.25	0.511	0.83	0.37, 1.89	0.658
Preoperative anemia	2.33	1.49, 3.63	<0.001	1.15	0.68, 1.96	0.597
Tumor location						
Lower third only	—	—		—	—	
Upper third/upper and middle third/whole stomach	2.71	1.46, 5.05	0.002	3.09	0.88, 10.9	0.078
Middle third/middle and lower third	1.17	0.70, 1.97	0.543	1.43	0.74, 2.77	0.284
Tumor size (cm)	1.42	1.29, 1.56	<0.001	1.29	1.13, 1.48	<0.001
Type of surgery						
Distal gastrectomy	—	—		—	—	
Total gastrectomy	1.99	1.19, 3.34	0.009	0.41	0.13, 1.28	0.125
Type of anastomosis						
Billroth I	—	—		—	—	
Billroth II	0.72	0.39, 1.34	0.302	0.59	0.29, 1.20	0.143
Roux en y	1.50	0.86, 2.61	0.151	0.79	0.41, 1.53	0.491
Level of lymphadenectomy						
D1+	—	—		—	—	
D2	0.76	0.31, 1.88	0.553	0.81	0.29, 2.29	0.696
D2+	2.97	1.04, 8.43	0.041	2.28	0.70, 7.37	0.170
Differentiation classification						
Well differentiated	—	—		—	—	
Moderately differentiated	2.16	0.66, 7.10	0.206	1.08	0.24, 4.78	0.921
Poorly differentiated	3.62	1.13, 11.6	0.030	1.62	0.36, 7.16	0.527
Signet ring cell	1.61	0.44, 5.96	0.473	0.87	0.17, 4.46	0.870
Invasive lymph nodes	4.04	2.41, 6.77	<0.001	2.13	1.22, 3.72	0.008
R0 surgery	0.77	0.11, 5.53	0.793	1.28	0.17, 9.58	0.812
T stage						
T1–T3	—	—		—	—	
T4a	8.41	3.68, 19.2	<0.001	3.95	1.63, 9.61	0.002
Complication						
No complication	—	—		—	—	
Minor complication	1.84	1.11, 3.04	0.017	1.40	0.81, 2.43	0.230
Major complication	2.17	1.04, 4.53	0.038	2.32	1.02, 5.30	0.045

Abbreviations: ASA‐PS, The American Society of Anesthesiologists physical status; BMI, body mass index; CI, confidence interval; HR, hazard ratio.

**TABLE 5:** Uni‐ and multivariable analyses of risk factors of disease‐free survival (excluded patients with preoperative chemotherapy).

	Univariable analysis			Multivariable analysis		
	HR	95% CI	*p*‐value	HR	95% CI	*p*‐value
Age (per 10 years increase)	1.11	0.96, 1.29	0.163	0.90	0.62, 1.31	0.593
Sex						
Male	—	—		—	—	
Female	1.24	0.88, 1.74	0.227	1.06	0.74, 1.53	0.739
BMI (kg/m^2^)	0.93	0.88, 0.99	0.027	0.96	0.90, 1.02	0.212
Charlson comorbidities Index	1.11	0.97, 1.26	0.128	1.17	0.80, 1.72	0.422
ASA‐PS classification						
I‐II	—	—		—	—	
III	1.15	0.69, 1.92	0.580	0.96	0.49, 1.86	0.899
Anemia	2.37	1.64, 3.42	<0.001	1.36	0.88, 2.11	0.164
Tumor location						
Lower third only	—	—		—	—	
Upper third/upper and middle third/whole stomach	2.78	1.64, 4.69	<0.001	2.37	0.90, 6.21	0.080
Middle third/middle and lower third	1.37	0.91, 2.06	0.129	1.57	0.92, 2.69	0.101
Tumor size (cm)	1.42	1.31, 1.53	<0.001	1.22	1.09, 1.37	<0.001
Type of surgery						
Distal gastrectomy	—	—		—	—	
Total gastrectomy	2.35	1.56, 3.53	<0.001	0.54	0.23, 1.26	0.156
Type of anastomosis						
Billroth I	—	—		—	—	
Billroth II	0.90	0.52, 1.56	0.719	0.78	0.42, 1.46	0.441
Roux en y	1.99	1.20, 3.30	0.008	1.17	0.65, 2.09	0.607
Level of lymphadenectomy						
D1+	—	—		—	—	
D2	0.64	0.33, 1.27	0.201	0.52	0.23, 1.14	0.100
D2+	1.95	0.83, 4.57	0.124	1.04	0.40, 2.69	0.938
Differentiation classification						
Well differentiated	—	—		—	—	
Moderately differentiated	2.35	0.84, 6.58	0.103	1.16	0.34, 3.94	0.806
Poorly differentiated	4.08	1.50, 11.2	0.006	1.67	0.49, 5.64	0.409
Signet ring cell	1.65	0.53, 5.11	0.386	0.81	0.21, 3.09	0.754
Invasive lymph nodes	4.02	2.62, 6.18	<0.001	2.33	1.46, 3.72	<0.001
R0 surgery	0.27	0.10, 0.74	0.011	0.40	0.14, 1.16	0.093
T stage						
T1–T3	—	—		—	—	
T4a	4.95	2.89, 8.47	<0.001	2.22	1.22, 4.03	0.009
Complication						
No complication	—	—		—	—	
Minor complication	1.72	1.11, 2.65	0.015	1.44	0.89, 2.32	0.141
Major complication	2.41	1.35, 4.31	0.003	2.63	1.37, 5.06	0.004

Abbreviations: ASA‐PS, The American Society of Anesthesiologists physical status; BMI, body mass index; CI, confidence interval; HR, hazard ratio.

## 4 DISCUSSION

Recently, LG became increasingly common for the surgical treatment of AGC due to its reported benefits, including reduced blood loss, less postoperative pain, and early return to normal bowel function. Our study demonstrated that LG for GC was safe and feasible. We observed an overall postoperative complications rate of 17.6%, a major complication rate of 5.3%, and a 30‐day postoperative mortality rate of 0.5%, which aligned with previous studies.^3^, ^4^, ^5^, ^7^, ^14^, ^18^, ^22^

Anastomotic leakage was a significant concern in gastrointestinal surgery and could lead to other complications such as bleeding, abdominal infection, peritonitis, and septic shock, which carried a risk of mortality. In our study, we observed an anastomotic and duodenal stump leak rate of 1.6%, in which two cases unfortunately resulted in septic shock and death. Our leakage rate was similar to recent studies^2^, ^5^, ^8^, ^9^, ^26^ and lower compared to earlier studies^27^, ^28^ possibly due to the advancements in surgical techniques and instrument technology. Pancreatic fistula was another common postoperative complication associated with significant inflammation and prolonged hospital stays. In our study, most pancreatic fistula cases were minor complications, with only one patient experiencing a major complication.

In our study, anastomosis related complications in Billroth I group was significantly higher than in Billroth II group. Notably, anastomotic tension due to the short remnant duodenum and the stomach might be a primary contributing reason. To mitigate this complication, Billroth I anastomosis should be performed in patients with the length of the remnant duodenum at least 2 cm. Additionally, suturing the intersection of the stapler lines is deemed necessary. Surgeons should be noted about the technique and the choice of reconstruction after distal gastrectomy to avoid anastomosis‐related complications.

Comprehending the pertinent risk factors is crucial for reducing the occurrence of postoperative complications. Our study identified several independent risk factors for postoperative complications. We found that BMI, Charlson comorbidities index, tumor location, tumor size, and advanced T stage were all independent risk factors for overall complications. Additionally, Charlson comorbidity index and type of anastomosis were identified as independent risk factors for major complications following LG. These findings were heterogenous to prior studies.^2^, ^7^, ^9^, ^21^, ^29^ Several studies indicated that postoperative complications were associated with operative time and blood loss (intraoperative bleeding).^2^, ^7^, ^9^, ^21^, ^29^ Contrarily, our study found these factors to have a non‐adverse impact. Advances in anesthesia techniques and enhancements in perioperative management have contributed to a reduction in complication rates among patients with unfavorable intraoperative conditions. It is worth mentioning that all comorbidities were effectively managed prior to surgery, and we implemented the ERAS program as a standard practice. Despite these measures, the Charlson comorbidities index still had an impact on the incidence of both overall and major postoperative complications, which is consistent with previous studies.^2^, ^9^, ^21^, ^29^

Numerous studies have investigated the relationship between postoperative complications and long‐term survival outcomes.^6^, ^8^, ^9^, ^13^, ^15^, ^16^, ^19^, ^27^, ^30^, ^31^, ^32^ Consistent with these findings, our study demonstrated that major complications had an impact on long‐term OS and DFS. It is crucial to closely monitor patients for potential major complications and take prompt action to prevent further morbidity and mortality. The negative association between major postoperative complications and poor survival after LG for GC can be attributed to the postoperative inflammatory response, which contributes to the host immunosuppression. This immunosuppression compromises cell‐mediated immunity, particularly affecting natural killer cells and cytotoxic T lymphocytes, thereby promoting the proliferation and metastasis of residual tumor cells.^33^ Additionally, major postoperative complications may result in lengthening postoperative hospital stay, delays or omission of adjuvant chemotherapy, further exacerbating the adverse impact on survival outcomes following LG for GC. In our study, the length of postoperative hospital stay was longer in the major complication group (average: 12 [10;19] days) compared to the no complication group (average: 7 [7;8] days). Moreover, the rate of patients receiving complete adjuvant chemotherapy was lower in the major complication group (55.6%) compared to the no complication group (68.4%). Previous research has showed that patients who received complete adjuvant chemotherapy had better survival outcomes compared to those who underwent gastrectomy alone.^34^

Major postoperative complications could significantly impact long‐term survival after laparosopic gastrectomy. However, other factors have also contributed to this scenario. Consistent with the findings of numerous previous studies,^6^, ^8^, ^9^, ^13^, ^15^, ^16^, ^19^, ^27^, ^30^, ^31^ our results also demonstrated that tumor size, the presence of invasive lymph nodes, and T4a stage were as important factors in predicting survival of both OS and DFS. In general, patients presenting with these tended to have lower survival rates and higher rates of postoperative complications compared to those with less advanced tumors.

In our study, the median length of follow‐up was more than 2.5 years. Therefore, estimates for survivals over 3 years are not certain. However, the Kaplan–Meier (Figure 2) showed the proportional hazard rates of the three groups (no complication, minor complication, and major complication). With longer follow‐up, we believe that the difference between groups still holds with statistical significance. Additionally, longer follow‐up is required to confirm this finding.

The study has several limitations that should be acknowledged. Firstly, this study was conducted at a single center, which may limit the generalizability of the findings to other settings or populations. Secondly, the relatively small number of specific complications observed in the study limited the ability to analyze the risk factors and the impact of each complication on survival outcomes. Further studies with larger sample sizes could provide more detailed insights into these relationships. Finally, the lack of information regarding the starting time of adjuvant chemotherapy limited the ability to fully analyze the association between postoperative complications and delayed initiation of adjuvant chemotherapy, which is known to affect survival outcomes.

In conclusion, the study provided evidence that LG was a safe and feasible surgical approach for GC. The incidence of major complications was found to be 5.3%. The Charlson comorbidities index was identified as an independent risk factor for both overall and major postoperative complications. Type of anastomosis was another risk factor for major complications. Importantly, major complications posed negative impact on survival outcomes. Therefore, efforts should be made to minimize the occurrence of complications and ensure timely and appropriate multimodal therapies for patients with complications to improve survival of GC patients.

## FUNDING INFORMATION

This research received no external funding.

## CONFLICT OF INTEREST STATEMENT

All authors declare no conflict of interests for this article.

## ETHICS STATEMENT

The study was conducted according to the guidelines of the Declaration of Helsinki and approved by Ethics Committee for Biomedical Research at the University Medical Center Ho Chi Minh City (registration number: 745/HDDD‐DHYD).

## Supporting information


Table S1.

## Data Availability

The data presented in this study are available on request from the corresponding author.
