# Supplementary material for: Risk factors of postoperative complications and their effect on survival after laparoscopic gastrectomy for gastric cancer
Source: Ann Gastroenterol Surg. 2024 Feb 24;8(4):580–94. doi: 10.1002/ags3.12780 (PMC11216791; doi:10.1002/ags3.12780)
Supplement: Supplementary file 1 — Table S1. [file AGS3-8-580-s001.docx]

# Supplementary Table S1. Postoperative complications

|  | **All patients (N=621)** | **Minor complication (N=76)** | **Major complication (N=33)** |
| --- | --- | --- | --- |
| Pneumonia | 28 (4.5) | 16 (21.1) | 12 (36.4) |
| Paralytic ileus | 21 (3.4) | 21 (27.6) | 0 (0.0) |
| Abdominal abscess | 17 (2.7) | 8 (10.5) | 9 (27.3) |
| Intra-abdominal bleeding | 14 (2.3) | 3 (3.9) | 11 (33.3) |
| Pancreatic leak | 14 (2.3) | 13 (17.1) | 1 (3.0) |
| Surgical site infection | 9 (1.4) | 5 (6.6) | 4 (12.1) |
| Anastomotic stenosis | 8 (1.3) | 4 (5.3) | 4 (12.1) |
| Pleural effusion | 5 (0.8) | 1 (1.3) | 4 (12.1) |
| Anastomotic leak | 5 (0.8) | 2 (2.6) | 3 (9.1) |
| Early bowel obstruction | 5 (0.8) | 2 (2.6) | 3 (9.1) |
| Duodenal stump leak | 5 (0.8) | 2 (2.6) | 3 (9.1) |
| In-hospital death | 3 (0.5) | 0 (0.0) | 3 (9.1) |
| Myocardial infarction | 3 (0.5) | 1 (1.3) | 2 (6.1) |
| Anastomotic bleeding | 2 (0.3) | 1 (1.3) | 1 (3.0) |
| Pulmonary edema | 1 (0.2) | 0 (0.0) | 1 (3.0) |
| Pulmonary embolism | 1 (0.2) | 0 (0.0) | 1 (3.0) |
| Ischemic stroke | 1 (0.2) | 0 (0.0) | 1 (3.0) |
| Chyle leak | 1 (0.2) | 1 (1.3) | 0 (0.0) |

*Statistics are n (%)*

# Supplementary Table S2. Kaplan-Meier estimates for survivals (excluded patients with preoperative chemotherapy)

|  | **12 months** | **24 months** | **36 months** | **48 months** | **60 months** |
| --- | --- | --- | --- | --- | --- |
| Overall survival |  |  |  |  |  |
| All patients | 96 (95, 98) | 90 (87, 92) | 84 (81, 88) | 79 (74, 83) | 74 (69, 80) |
| No complication | 97 (96, 99) | 91 (88, 94) | 86 (82, 90) | 81 (77, 86) | 78 (73, 84) |
| Minor complication | 94 (89, 100) | 86 (78, 95) | 77 (67, 88) | 70 (59, 83) | 61 (48, 77) |
| Major complication | 87 (76, 100) | 79 (65, 96) | 79 (65, 96) | 66 (48, 90) | 66 (48, 90) |
| Recurrence-free survival |  |  |  |  |  |
| All patients | 90 (88, 93) | 84 (80, 87) | 76 (72, 80) | 71 (67, 76) | 69 (64, 74) |
| No complication | 92 (89, 94) | 85 (82, 89) | 78 (74, 83) | 75 (70, 79) | 73 (68, 78) |
| Minor complication | 86 (79, 95) | 80 (71, 90) | 67 (56, 80) | 60 (49, 75) | 55 (43, 70) |
| Major complication | 83 (71, 98) | 64 (49, 85) | 61 (45, 82) | 50 (34, 75) | 50 (34, 75) |

# *Statistics are Kaplan-Meier estimate (95% confidence interval)*

# Supplementary Table S3. Type of recurrences

|  | **All patients (N=104)** | **No complication (N=78)** | **Minor complication (N=17)** | **Major complication (N=9)** |
| --- | --- | --- | --- | --- |
| Recurrence type |  |  |  |  |
| Locoregional | 10 (9.6) | 6 (7.7) | 2 (11.8) | 2 (22.2) |
| Hematogenous | 15 (14.4) | 11 (14.1) | 4 (23.5) | 0 (0.0) |
| Peritoneal | 62 (59.6) | 47 (60.3) | 10 (58.8) | 5 (55.6) |
| Distant lymph nodes metastasis | 9 (8.7) | 8 (10.3) | 0 (0.0) | 1 (11.1) |
| Mixed | 8 (7.7) | 6 (7.7) | 1 (5.9) | 1 (11.1) |

*Statistics are n (%)*
